# Supplementary material for: Efficacy of hyperthermic intraperitoneal chemotherapy in colorectal cancer: A phase I and III open label randomized controlled registry-based clinical trial protocol
Source: PLoS One. 2024 Mar 4;19(3):e0294018. doi: 10.1371/journal.pone.0294018 (PMC10911585; doi:10.1371/journal.pone.0294018)
Supplement: S1 File — (DOCX) [file pone.0294018.s001.docx]

CLINICAL STUDY PROTOCOL

EFFIPEC – Efficacy of Hyperthermic Intraperitoneal Chemotherapy
Single-arm Phase I study, followed by an open-label, randomized, controlled
registry-based phase III trial

| **Sponsor Project No:** | EFFIPEC |  |
| --- | --- | --- |
| **EudraCT number:** | 2020-005210-18 |  |
| **Investigational Product:** | 5-Fluorouracil and Irinotecan |  |
|  |  |  |
| **Sponsor:** | Uppsala University |  |
| **Sponsor Representative:** | Peter Cashin, MD, PhD  Telephone: +46 (0)18-617 43 04  E-mail: peter.cashin@surgsci.uu.se |  |
| **Coordinating Investigator:** | Peter Cashin, MD, PhD |  |
| **Investigators:** | Lana Ghanipour, MD, PhD (PI)  Ingvar Syk, MD, PhD (PI)  Elinor Bexe Lindskog, MD, PhD (PI)  Gabriella Jansson Palmer, MD, PhD (PI) |  |
|  |  |  |
| **The clinical study will be conducted, and essential documentation archived, in compliance with relevant UCR SOPs and standards, which incorporate the requirements of the ICH Guideline for Good Clinical Practice.** | | |

| **SYNOPSIS** | | |
| --- | --- | --- |
| Title of study:   EFFIPEC – Efficacy of Hyperthermic Intraperitoneal Chemotherapy (HIPEC) | | |
| Name of Sponsor/Company: Uppsala University/Peter Cashin | | |
| Name of investigational product: 5-Fluorouracil and Irinotecan | | |
| Investigator(s) and study center(s): |  | |
| Peter Cashin MD, PhD & Lana Ghanipour, MD, PhD | Uppsala University Hospital | |
| Ingvar Syk, MD, PhD | Skåne University Hospital | |
| Elinor Bexe Lindskog, MD, PhD | Sahlgrenska University Hospital | |
| Gabriella Jansson Palmer, MD, PhD  Pending approval  Pending approval | Karolinska University Hospital  Aarhus University Hospital, Denmark  Oslo University Hospital, Norway | |
| Planned study period:  2021-01-01 to 2029-12-31 | | Phase of development:  Phase I/III |
| Objectives:  Primary objectives:  Phase I: To determine the maximum tolerated dose of 5-Fluorouracil 24-hour EPIC, in combination with intensified oxaliplatin/irinotecan HIPEC, for treatment of colorectal cancer.  Phase III: To study the recurrence-free survival (RFS) of patients 12 months after treatment  Secondary objectives:  Overall and recurrence-free survival up to 5 years  Postoperative complication rates within 30 days  Quality of life of patients up to 3 years after study treatment | | |
| Methodology:  A combined phase I/III trial, consisting of an initial dose titration study followed by an open-label, randomized controlled study. | | |
| Number of subjects (planned):  Dose escalation study: min 15 and max 30 patients - Number of patients is based on toxicity observed.  Efficacy study: 326 patients. Interim analysis is done after 140 patients are included | | |
| Diagnosis:  Colorectal cancer | | |
| Inclusion criteria:   1. Provision of written informed consent prior to any study specific procedures. 2. ECOG Performance Status Score 0,1 or 2 alternatively Karnofsky 60-100 3. Adequate kidney, liver, bone marrow function according to laboratory tests 4. For females of childbearing potential, a negative pregnancy test must be documented 5. ≥ 18 years old and <75 years old 6. Colorectal cancer with peritoneal metastases +/- liver metastases (maximum 3) 7. All patients deemed eligible for CRS and HIPEC according to clinical routine management during a HIPEC multidisciplinary board at each respective hospital can be included. | | |
| Exclusion criteria:   1. Previous severe toxicity/allergic reactions to systemic chemotherapy agents oxaliplatin or irinotecan or 5-fluorouracil 2. Unable to tolerate intensified HIPEC treatment due to comorbidity 3. Metastasis other than peritoneum or liver 4. Complex liver-perenchymal sparing surgery or hemihepatectomy procedures are to be excluded. 5. Previous CRS or HIPEC 6. Pregnant or lactating (nursing) women 7. Active infections requiring antibiotics 8. Active liver disease with positive serology for active hepatitis B, C, or known HIV 9. Concurrent administration of any cancer therapy other than planned study treatment within 4 weeks prior to and up to 4 weeks after study treatment 10. Incomplete cytoreduction defined as completeness of cytoreduction score 1-3 11. Histopathology of other origin than colorectal cancer | | |
| Investigational product, dosage and mode of administration:  Injection of irinotecan 360 mg/m^2^ and 5-fluorouracil 24-hr EPIC 250-850 mg/m^2^ in combination with Oxaliplatin 360 mg/m^2^ and an intraoperational IV of 5-fluorouracil bolus 400 mg/m^2^ with calcium folinate 60mg/m^2^.  The EPIC treatment is given after the abdomen is completely sutured in the operating theatre. The dose will be divided equally into 2 injections á 200ml each through two abdominal drains. | | |
| Duration of treatment:  24 hours | | |
| Active control, dosage and mode of administration:  Injection of oxaliplatin 460 mg/m^2^ and an intraoperational IV of 5-fluorouracil 400 mg/m^2^, and calcium folinate 60 mg/m^2^ | | |
| Criteria for evaluation:  Safety: Safety will be assessed by the Clavien-Dindo classification and reporting of Adverse Events.  The maximum tolerated dose of 5-FU will be determined by giving the first 15-30 patients escalating doses of 5-FU. The morbidity of each dose level of 5-FU 24-hour EPIC will be evaluated by the Data Monitoring Committee 30 days post-treatment. A dose level with ≤1/3 or ≤2/6 with 3b-4 Clavien-Dindo morbidity may proceed to the next dose level. Following the dose titration study, all patients will receive the maximum dose of 5-FU. An interim safety evaluation will be performed after the first 140 patients have been randomized and treated with HIPEC or HIPEC in combination with EPIC. The Clavien-Dindo grade 3-5 morbidity is expected to be approximately 0.35 (35%). If the experimental arm’s 95% CI falls within 0.4 or below, the study will be allowed to continue.  Efficacy: Recurrence free survival (RFS) is determined at 12-months post treatment through the HIPEC registry. Postoperative complications rates with-in 30 days, 5-year overall survival and RFS are further determined through the HIPEC registry and quality of life is assessed by questionnaires through the QoL registry. | | |
| **Dosage of Investigational Products:**  The maximum tolerated dose (MTD) of 5-FU will be determined by giving the first 15-30 patients escalating doses of 5-FU. The morbidity of each dose level of 5-FU 24-hour EPIC will be evaluated by the Data Monitoring Committee within 30 days post-treatment. A dose level with ≤1 of 3 patients or ≤2of 6 patients (extended group size) with 3b-5 morbidity may proceed to the next dose level. If the frequency of 3b-5 morbidity is higher, MTD has been passed and the dose of 5-FU in the efficacy study will be the next lower dose level. If MTD is not reached the highest dose level of 5-FU (850 mg/m^2^) will be used. | | |
| Statistical methods:  Recurrence free and overall survival of the two arms will be compared using the parameters: treatment arm, age, PCI, CC score, use of systemic perioperative chemotherapy (neoadjuvant and/or adjuvant), concomitant liver metastases resection, colon or rectal primary tumor, and lymph-node metastasized primary tumor.  QoL will be analyzed by comparing the answers to questions 29 and 30 in QLQ-C30 by Mann-Whitney U test and Freidman’s repeated measures ANOVA test. Specifically, differences between the sexes will be investigated.  Statistical significance will be set at p=0.05. | | |

# Table of contents

Page

[1 Table of contents 5](#_Toc62115096)

[2 List of abbreviations AND DEFINITIONS OF TERMS 8](#_Toc62115097)

[3 GENERAL INFORMATION/study administrative structure 9](#_Toc62115098)

[4 SIGNATURE PAGE 12](#_Toc62115099)

[5 BACKGROUND INFORMATION 13](#_Toc62115100)

[5.1 Investigational drugs 13](#_Toc62115101)

[5.2 Disease background and preliminary efficacy 13](#_Toc62115102)

[5.3 Justification of administration route 14](#_Toc62115103)

[5.4 Justification of Study design 14](#_Toc62115104)

[5.5 Study population 15](#_Toc62115105)

[5.6 Compliance with GCP and regulatory requirements 15](#_Toc62115106)

[6 STUDY OBJECTIVES 15](#_Toc62115107)

[6.1 Primary objectives 15](#_Toc62115108)

[6.1.1 Phase I 15](#_Toc62115109)

[6.1.2 Phase III 15](#_Toc62115110)

[6.2 secondary objectives 16](#_Toc62115111)

[7 ENDPOINTS 16](#_Toc62115112)

[7.1 Primary Endpoint 16](#_Toc62115113)

[7.1.1 Phase I 16](#_Toc62115114)

[7.1.2 Phase III 16](#_Toc62115115)

[7.2 Secondary Endpoints 16](#_Toc62115116)

[8 STUDY DESIGN 16](#_Toc62115117)

[8.1 Study outline 16](#_Toc62115118)

[8.2 Study assessments and procedures 17](#_Toc62115119)

[8.2.1 Dose titration 17](#_Toc62115120)

[8.2.2 Efficacy study 18](#_Toc62115121)

[8.3 Schedule of events 19](#_Toc62115122)

[9 SELECTION AND WITHDRAWAL OF SUBJECTS 20](#_Toc62115123)

[9.1 Subject inclusion criteria 20](#_Toc62115124)

[9.2 Subject exclusion criteria 20](#_Toc62115125)

[9.3 Withdrawal of subjects 20](#_Toc62115126)

[9.4 Monitoring subject compliance 20](#_Toc62115127)

[10 TREATMENT OF SUBJECTS 21](#_Toc62115128)

[10.1 Treatment administration 21](#_Toc62115129)

[10.2 Description of investigational products 22](#_Toc62115130)

[10.3 Packaging and labelling of investigational products 22](#_Toc62115131)

[10.4 Storage and handling 22](#_Toc62115132)

[10.5 Randomisation and blinding 23](#_Toc62115133)

[10.6 Concomitant therapy 23](#_Toc62115134)

[10.7 Accountability of investigational products 23](#_Toc62115135)

[11 ASSESSMENT OF EFFICACY 23](#_Toc62115136)

[11.1 Clinical and laboratory assessments 24](#_Toc62115137)

[11.1.1 Timing and method of the assessment 24](#_Toc62115138)

[12 ASSESSMENT OF SAFETY 25](#_Toc62115139)

[12.1 Timing and methods for Assessing safety parameters 25](#_Toc62115140)

[12.2 Definition, assessment and causality of adverse events 26](#_Toc62115141)

[12.3 Definition of serious adverse events 26](#_Toc62115142)

[12.4 DATA MOnitoring committee 26](#_Toc62115143)

[12.5 Safety parameters 27](#_Toc62115144)

[12.5.1 Interim safety analysis of the Efficacy study 27](#_Toc62115145)

[12.6 Reporting of adverse events, serious adverse events, AND suspected unexpected serious adverse reactions 28](#_Toc62115146)

[12.7 Follow-up of adverse events and serious adverse events 28](#_Toc62115147)

[12.8 ANNUAL Development safety update report (DSUR) 28](#_Toc62115148)

[12.9 Procedures in case of medical emergency 28](#_Toc62115149)

[13 STATISTICS AND DATA MANAGEMENT 29](#_Toc62115150)

[13.1 Data management 29](#_Toc62115151)

[13.2 Statistical analysis 30](#_Toc62115152)

[13.2.1 Statistical methods 30](#_Toc62115153)

[13.3 Determination of sample size 32](#_Toc62115154)

[13.4 Statistical significance 32](#_Toc62115155)

[13.5 Criteria for the termination of the trial 32](#_Toc62115156)

[13.6 Missing data management 33](#_Toc62115157)

[13.7 Intention-to-treat analysis 33](#_Toc62115158)

[14 Direct access to source data/documents 33](#_Toc62115159)

[15 Quality control and quality assurance 33](#_Toc62115160)

[15.1 Source data 33](#_Toc62115161)

[15.2 Monitoring 34](#_Toc62115162)

[16 ETHICS 34](#_Toc62115163)

[16.1 Ethical review authority 34](#_Toc62115164)

[16.2 Ethical Conduct of the study 35](#_Toc62115165)

[16.3 Risk - benefits 35](#_Toc62115166)

[16.3.1 Covid-19 37](#_Toc62115167)

[16.4 Patient information and informed consent 37](#_Toc62115168)

[17 Data handling and record keeping 38](#_Toc62115169)

[17.1 Case Report Forms 38](#_Toc62115170)

[17.2 Record keeping 38](#_Toc62115171)

[18 INSURANCE 39](#_Toc62115172)

[19 PUBLICATION POLICY 39](#_Toc62115173)

[20 SUPPLEMENTS 39](#_Toc62115174)

[20.1 Changes of the study protocol 39](#_Toc62115175)

[20.2 Application to regulatory authorities 39](#_Toc62115176)

[20.3 Staff information 39](#_Toc62115177)

[20.4 Criteria for termination of the study 39](#_Toc62115178)

[20.5 Study timetable 40](#_Toc62115179)

[21 REFERENCES 41](#_Toc62115180)

[22 signed agreement of the study protocol 43](#_Toc62115181)

[23 Appendices 44](#_Toc62115182)

[23.1 Declaration of Helsinki 44](#_Toc62115183)

[23.2 serious adverse event report form 44](#_Toc62115184)

# List of abbreviations AND DEFINITIONS OF TERMS

5-FU 5-fluorouracil

AE Adverse Event

CIOMS Council for International Organizations of Medical Sciences

CRCPM ColoRectal Cancer with Peritoneal Metastases

CRF Case Report Form

CRS Cytoreductive surgery

CC score Completeness of cytoreduction score

DCF Data Clarification Form

DMC Data Monitoring Committee

EPIC Early Postoperative Intraperitoneal Chemotherapy

EOS End of study

ERA Ethical Review Authority

G-CFS Granulocyte Colony Stimulating Factor

GCP Good Clinical Practice

HIPEC Hyperthermic IntraPEritoneal Chemotherapy

IEC Independent Ethics Committee

IMP Investigational Medicinal Product

MPA Medical Products Agency

PI Principal Investigator

QoL Quality of Life

RFS Recurrence-Free Survival

SAE Serious Adverse Event

SUSAR Unexpected Serious Adverse Reaction

UCR Uppsala Clinical Research Center

# GENERAL INFORMATION/study administrative structure

| **Sponsor representative/**  **Coordinating investigator** | Peter Cashin, MD, PhD  Uppsala University Hospital  Ing 70, Akademiska sjukhuset,  75185 Uppsala, Sweden  Telephone: +46 (0)18 6174304  E-mail: peter.cashin@surgsci.uu.se |
| --- | --- |
| **Site Principal Investigator** | Lana Ghanipour, MD, PhD  Uppsala University Hospital  Ing 70, Akademiska sjukhuset,  75185 Uppsala, Sweden  Telephone: +46 (0)18 6110000  E-mail: lana.ghanipour@surgsci.uu.se |
| **Site Principal Investigator** | Ingvar Syk, MD, PhD  Skåne University Hospital  20502 Malmö Sweden  Telephone: +46 (0) 40 331000  E-mail: [ingvar.syk@med.lu.se](mailto:ingvar.syk@med.lu.se) |
| **Site Principal Investigator** | Elinor Bexe Lindskog, MD, PhD  Sahlgrenska University Hospital,  416 85 Göteborg, Sweden  Telephone: +46 (0) 31 3421000  elinor.bexe-lindskog@surgery.gu.se |
| **Site Principal Investigator** | Gabriella Jansson Palmer, MD PhD  Department of molecular medicin and surgery, Karolinska Institutet and Department of pelvic cancer, Gastrointestinal oncology and colorectal surgical unit, Karolinska University Hospital, Stockholm, Sweden  Telephone: +46 (0) 8 517 700 00  E-mail: gabriella.palmer@ki.se |
| **International site investigators**  **Data manager** | Pending approval – Se separate appendix  Aarhos University Hospital, Denmark  Pending approval – Se separate appendix  Oslo University Hospital, Norway  Christopher Lagerros  Lagerros IT AB  Hammerstavägen 79, 122 60 Enskede, Sweden  Telephone: +46-(0)733 249460  Email: chrille@lagerros.se |
| **Statistician** | Patrik Öhagen  Uppsala Clinical Research Center  Dag Hammarskjölds Väg 38, 751 85 Uppsala, Sweden  Telephone: +46 (0)18 611 13 90  Email: patrik.ohagen@ucr.uu.se |
| **Medical expert** | Jan-Erik Frödin, MD, PhD  Senior clinical oncologist  Department of oncology and pathology, Karolinska Institutet and Department of pelvic cancer, Gastrointestinal oncology and colorectal surgical unit, Karolinska University Hospital, Stockholm, Sweden Telephone: +46 (0) 8 517 700 00  [Jan-erik.frodin@karolinska.se](mailto:Jan-erik.frodin@karolinska.se) |
| **Contract Research Organisation** | Uppsala Clinical Research Center  Dag Hammarskjölds Väg 38, 751 85 Uppsala, Sweden |
| **Site Co-investigators** | Skåne University Hospital:  Jenny Brändstedt, MD, PhD  Karolinska University Hospital:  Caroline Nordenvall, MD,PhD  Sahlgrenska University Hospital:  Dan Asplund, MD, PhD |
| **Data Monitoring Committee** | 1: DMC Chairperson  Hans Hagberg, MD, PhD, Docent  Department of Oncology  Uppsala University Hospital  Telephone: +46 (0)18-611 0000  E-mail: Hans.Hagberg@igp.uu.se  2: Member  Peter Stålberg MD, Prof  Institute of Surgical Sciences  Endocrine Surgery  Uppsala University  Telephone: +46 (0)18-611 0000  E-mail: Peter.Stalberg@surgsci.uu.se  3: Member  Kjersti Flatmark MD, Prof  Colorectal Surgery Unit  Oslo University Hospital |
|  | Telephone: +47 (0)91 16 84 66  E-mail: kjersti.flatmark@rr-research.no |
|  |  |

# SIGNATURE PAGE

**SPONSOR REPRESENTATIVE**

| Peter Cashin, MD, PhD  Uppsala University  Ing 70, Akademiska sjukhuset,  751 85, Uppsala, Sweden | |
| --- | --- |
| **Signature and date:** | 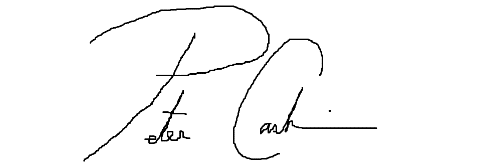  2022-07-01  **_______________________________________** |

**CO-ORDINATING Investigator**

I agree to conduct the study according to this protocol and according to the ethical principles that have their origin in the Declaration of Helsinki and are consistent with Good Clinical Practice and the applicable national laws and regulations.

| Peter Cashin, MD, PhD  Uppsala University  Ing 70, Akademiska sjukhuset,  75185 Uppsala, Sweden | |
| --- | --- |
| **Signature and date:** | **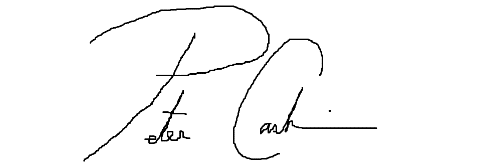**  2022-07-01  **_______________________________________** |

# BACKGROUND INFORMATION

## Investigational drugs

Irinotecan is an antineoplastic enzyme inhibitor primarily used in the treatment of colorectal cancer. It is a derivative of camptothecin that inhibits the action of topoisomerase I. 5-fluorouracil (5-FU) is a nucleobase analogue that is uracil in which the hydrogen at position 5 is replaced by fluorine. It is an antineoplastic agent which acts as an antimetabolite - following conversion to the active deoxynucleotide, it inhibits DNA synthesis (by blocking the conversion of deoxyuridylic acid to thymidylic acid by the cellular enzyme thymidylate synthetase) and thereby slows tumour growth. Both drugs are well known as systemic chemotherapy treatments of colorectal cancer. The side-effects are also well known and well-studied for many years. This trial is investigating a new combination treatment for colorectal cancer with peritoneal metastases. It is a treatment administered at the time of surgery.

## Disease background and preliminary efficacy

Colorectal cancer with peritoneal metastases (CRCPM) is now routinely treated with cytoreductive surgery (CRS) and hyperthermic intraperitoneal chemotherapy (HIPEC). A recent study, PRODIGE 7, has cast some doubt on the efficacy of oxaliplatin-based HIPEC for CRCPM [1, 2]. Due to some limitations in the PRODIGE 7 study, follow-up trial discussions have been difficult. However, there is a proof of concept from both gastric cancer and ovarian cancer (relatively chemoresistant [gastric] and chemosensitive solid tumors [ovarian]) that CRS +/- HIPEC has shown a benefit for patients treated with HIPEC [3, 4]. Thus, it seems clinically relevant and meaningful to continue the search for a HIPEC treatment that is effective in colorectal cancer. In two recent studies, there is a disease-free survival benefit in favor of oxaliplatin/irinotecan HIPEC vs single drug oxaliplatin HIPEC [5, 6]. Combination or intensified HIPEC treatment leads to increased recurrence-free survival (RFS), particularly peritoneal recurrence-free survival. Figure 1 demonstrates a significant benefit with each step of HIPEC intensification [5].

**Figure 1 [6]** – Colorectal cancer with peritoneal metastases treated with HIPEC +/-EPIC. The blue line represents the least intense perioperative intraperitoneal chemotherapy treatment, while the green and red lines represent stepwise intensified perioperative intraperitoneal chemotherapy treatment.

## Justification of administration route

The concept of both HIPEC and early postoperative intraperitoneal chemotherapy (EPIC) is to maximize the effect of chemotherapy on the peritoneum in order to prevent locoregional recurrence. The surgical treatment (cytoreductive surgery) is aimed at removing all visible tumor tissue from the peritoneum. The aim of the perioperative chemotherapy treatments of HIPEC and EPIC is then to eradicate potentially remaining microscopic peritoneal disease. It is well-known that the chemotherapy distribution from the bloodstream to the peritoneal cavity is limited due to the blood-peritoneal barrier. Therefore, this trial is using two intraperitoneal chemotherapy treatment options instead of systemic chemotherapy.

## Justification of Study design

The reason for going from a phase I to III combined approach is that the triple drug treatment (oxaliplatin, irinotecan, and 5-FU) has already been used in the medical oncology clinic as a routine clinical treatment for many years with a well-known and reported toxicity profile; and it has also, in some countries, been used in the HIPEC setting with 5-days of 5-FU EPIC. The initial plan was to do a direct phase III study. However, since it is planned to give a single dose of 5-FU instead of 5 daily treatments, it will probably be possible to increase the concentration of 5-FU EPIC. As such, the study committee opted to do a dose-titration study (phase I) including 15-30 patients to evaluate the possibility of using a slightly higher single dose 24-hour 5-FU. Instead of a phase II trial, an interim analysis after treatment of the first 140 patients in phase III was added to evaluate safety and aim for a phase III final comparison including 326 patients.

The rationale behind two investigational drugs (irinotecan and 5-FU) is that adding only one drug is not enough to provide a synergy that can produce a reasonable investigational clinical effect (see figure 1). The dose-titration of irinotecan to oxaliplatin HIPEC has already been performed [14]. Thus, the phase I part of the trial is investigating the addition of 5-FU EPIC in a dose-titration manner to oxaliplatin + irinotecan HIPEC. The phase III part of the trial requires both drugs to be added for efficacy reasons and it is not feasible clinically to have several trial arms. Since the triple drug combination has already been in use in France with cytoreductive surgery and the combination is well studied and in use in clinical routine medical oncology treatment for metastasized colorectal cancer, it is the synergy of all three drugs combined with cytoreductive surgery that is being studied in phase III. Hence, all conclusions will be related to the combination treatment as such.

The risks of adding 5-FU EPIC to HIPEC has been reviewed already in several trials [7]. The risk of morbidity, reoperation, or postop mortality is not increased [6]. Although, one particular side-effect of neutropenia is expected to increase, it does not increase the risk of other morbidity [8]. In order to offset this side-effect, a planned bone marrow stimulation treatment will be administered.

## Study population

The trial concerns patients with colorectal cancer and peritoneal metastases. The total number of patients possibly eligible for the study will be about 120 patients per year in Sweden.

## Compliance with GCP and regulatory requirements

This study will be conducted in compliance with this protocol and in accordance with the Good Clinical Practice guidelines (ICH-GCP), the principles of the Declaration of Helsinki and according to applicable regulatory regulations and directives.

The study will not start until the protocol has been approved by the Ethical Review Authority (Etikprövningsmyndigheten) and the Medical Products Agency (Läkemedelsverket).

# STUDY OBJECTIVES

## Primary objectives

### Phase I

Phase I: To determine the maximum tolerated dose of 5-Fluorouracil 24-hour EPIC, in combination with intensified oxaliplatin/irinotecan HIPEC, for treatment of colorectal cancer.

### Phase III

## Phase III: To study the recurrence-free survival (RFS) of patients 12 months after treatment. secondary objectives

1: Overall and recurrence-free survival up to 5 years,

2: Postoperative complication rates within 30 days

3: Quality of life of patients up to 3 years after study treatment

# ENDPOINTS

## Primary Endpoint

### Phase I

- Maximum tolerated dose of 5-FU 24-hour EPIC. Possible toxicity will be reported as Adverse Events.

### Phase III

- Recurrence-free survival at 12 months.

## Secondary Endpoints

The secondary endpoints are to evaluate the:

- Overall survival and RFS up to 5 years through the HIPEC registry
- Postoperative complication rates within 30 days
- Quality of life of patients up to 3 years after treatment

# STUDY DESIGN

## Study outline

A dose titration study and a combined superiority registry-based open-label randomized control trial is planned to answer the trial objectives. The study will be registry-based to allow simpler and more comprehensive follow-up. Patients with colorectal cancer will be treated with cytoreductive surgery (CRS) together with either standard oxaliplatin HIPEC (the control for the efficacy study) or oxaliplatin/irinotecan HIPEC in combination with 5-FU 24-hour EPIC. The 5-FU will be administered postoperatively when the abdomen is completely sutured. The drug is divided equally into 2 injections of 200 ml each and injected through two abdominal drains that are clamped for 16hours.

For dose escalation, the titration groups (á 3 or 6 patients) are followed for 30 days postoperatively after which the Data Monitoring Committee (DMC) will determine whether or not to increase the 5-FU dose for the following group of patients.

To study efficacy, randomization is performed intraoperatively. The patient is followed up postoperatively for a total of 3 years for the secondary endpoints which may be extended by the study committee to 5 years. Since the trial is registry based, the long-term follow-up does not require separate eCRF evaluations. These evaluations can be automatically retrieved from the registry – both recurrence data, quality of life, and morbidity data. Some specific eCRF evaluations will be integrated as a separate study part of the HIPEC registry, such as inclusion/exclusion criteria and adverse event reporting (including SUSAR reporting).

## Study assessments and procedures

### Dose titration

A single-arm dose titration trial (Table 1). Sample size: minimum 15 patients (3 patients per dose level) – maximum 30 (6 patients per dose level) patients.

Table 1. Dose escalation of 5-FU 24-hour EPIC for each titration level and concomitant treatment where irinotecan is also an investigational drug (the other drugs are non-investigational and given to both groups in the randomized part of the trial).

| Level | Non-investigational  Oxaliplatin | **Investigational**  **Irinotecan** | **Investigational**  **5-FU**  **24-hour EPIC** | Non-investigational  5-FU  bolus | Non-investigational  Calcium folinate |
| --- | --- | --- | --- | --- | --- |
| -1 | 360 mg/m^2^ | **360 mg/m^2^** | **250 mg/m^2^** | 400 mg/m^2^ | 60mg/m^2^ |
| 0 | 360 mg/m^2^ | **360 mg/m^2^** | **400 mg/m^2^** | 400 mg/m^2^ | 60mg/m^2^ |
| 1 | 360 mg/m^2^ | **360 mg/m^2^** | **550 mg/m^2^** | 400 mg/m^2^ | 60mg/m^2^ |
| 2 | 360 mg/m^2^ | **360 mg/m^2^** | **700 mg/m^2^** | 400 mg/m^2^ | 60mg/m^2^ |
| 3 | 360 mg/m^2^ | **360 mg/m^2^** | **850 mg/m^2^** | 400 mg/m^2^ | 60mg/m^2^ |

Endpoint is Clavien-Dindo morbidity classification 3b-5 (Table 2). All patients will be reviewed at each of the dose levels by the DMC prior to allowing the next group of patients to advance to the following level. The toxicity follow-up is 30 days if no incidents occur. If the postoperative rehabilitation is slower or incidents occur, the follow-up period may be extended to by the site Principal Investigator or Co-investigator.

If none or one of the first three patients at a dose level has a grade 3b-4b the DMC will review the data and permit the start of the next level. If more than one patient experiences a grade 3b-4b another three patients will be included at the same dose level before the DMC will review the data. A frequency of 3b-4b morbidity in ≤1 of 3 or ≤2 of 6 patients may result in a recommendation by the DMC to proceed to the next dose level. A higher frequency of grade 3b-4b means the maximum tolerated dose (MTD) has been passed, and the dosage used in the efficacy study will be the nearest underlying dose level. Any grade 5 event pauses all inclusion for review. Depending on cause, the trial may continue after DMC review.

Table 2. Clavien-Dindo classification.

| Grades | Definition |
| --- | --- |
| Grade 1 | Any deviation from the normal postoperative course without the need for pharmacological treatment or surgical, endoscopic and radiological interventions Allowed therapeutic regimens are drugs as antiemetics, antipyretics, analgesics, diuretics and electrolytes and physiotherapy. This grade also includes wound infections opened at the bedside. |
| Grade 2 | Requiring pharmacological treatment with drugs other than such allowed for grade 1 complications. Blood transfusions and total parenteral nutrition are also included. |
| Grade 3 | Requiring surgical, endoscopic or radiological intervention |
| -3a | Intervention not under general anaesthesia |
| -3b | Intervention under general anaesthesia |
| Grade 4 | Life-threatening complication (including CNS complications) requiring IC/ICU-management |
| -4a | Single organ dysfunction (including dialysis) |
| -4b | Multi-organ dysfunction |
| Grade 5 | Death of a patient |

### Efficacy study

Figure 2: Flowchart for the efficacy study

| All colorectal cancers with peritoneal metastases | | | | |
| --- | --- | --- | --- | --- |
| ↓  Randomization intraoperatively  ↙ ↘ | | | | |
| **A: Standard HIPEC**  **(70 patients)**   1. Oxaliplatin  - 460 mg/m^2^ (HIPEC)  1. 5-fluorouracil  - 400 mg/m^2^ IV bolus (Intraop)  1. Calcium folinate  - 60 mg/m^2^ IV (Intraop) | |  | **B:** **Intensified HIPEC+EPIC**  **(70 patients)**   1. Oxaliplatin    - 360 mg/m^2^ (HIPEC) 2. Irinotecan    - 360 mg/m^2^ (HIPEC) 3. 5-FU Bolus    - 400 mg/m^2^ IV (Intraop) 4. Calcium folinate 5. 60 mg/m^2^ IV (Intraop) 6. 5-FU 24-hr EPIC  - According to the dose titration study | |
|  | |  | Prophylactic G-CSF postop day 4-8 | |
| ↓ |  | | | ↓ |
| Interim safety evaluation:  Grade 3-5 Clavien-Dindo morbidity comparison | | | | |
| ↓ | | | | |
| **A: Standard HIPEC**  **(163 patients)** | |  | **B:** **Intensified HIPEC+EPIC**  **(163 patients)** | |
| ↓ | |  | ↓ | |
| Primary endpoint:   - 12-month RFS   Secondary endpoints:   - Overall survival and RFS up to 5 years: Through the HIPEC registry - QoL: Through the QoL registry - Morbidity: Through the HIPEC registry | | | | |

## Schedule of events

Table 3. Schedule of investigational events.

|  | Preop Visit | Day of treat-ment | 1  In-hospital | 2  In-hospital | 3  Out-patient | 4  Out-patient | 5  Out- patient | 6  Out-patient | 7  EOS |
| --- | --- | --- | --- | --- | --- | --- | --- | --- | --- |
|  | -1m | 0 d | 5 d ± 2 | 10 d ± 3 | 30 d +2m | 90 d  ±4m | 1y ±3m | 3 y ±3m | 5 y ±3m |
| Physical examination  (eCRF follow-up) | x |  | x | x |  |  |  |  |  |
| Telephone follow-up  (eCRF follow-up) |  |  |  |  | x | x | x | x |  |
| eCRF Inclusion/screening | x |  |  |  |  |  |  |  |  |
| Signed informed consent | x |  |  |  |  |  |  |  |  |
| Randomization* |  | x* |  |  |  |  |  |  |  |
| CRS + HIPEC/EPIC treatment data  HIPEC registry treatment section |  |  |  |  |  | x |  |  |  |
| eCRF Drugs – medication/chemotherapy** | x |  |  |  |  | x |  |  |  |
| CT scan |  |  |  |  |  |  | x |  |  |
| eCRF Blood work*** |  |  |  |  | x |  |  |  |  |
| Questionnaire (QLQ-C30, CR29, STO22) HIPEC registry acquired | x |  |  |  |  | x | x | x |  |
| Morbidity from HIPEC registry (incl. eCRF AER/eCRF follow-up) |  |  | x | x | x |  |  |  |  |
| Overall survival HIPEC registry§ |  |  |  |  |  |  |  |  | x |
| Recurrence free survival HIPEC registry |  |  |  |  |  |  | x | x | x |

* only after the maximum tolerated dose of 5-FU has been determined

** (-1m) – previous chemotherapy and current medications, (90d) adjuvant chemotherapy administered

*** CEA, CA 19-9, CA 125 are taken preoperatively (-1m). Haemoglobin, C-reactive protein (CRP), white blood cell count (WBC), neutrophil count, platelet count, albumin, creatinine, alanine transaminase (ALT) are taken preoperatively and postoperatively 5 times during the first 10 days. The lowest haemoglobin, highest CRP, lowest neutrophil and WBC, lowest platelet count, lowest albumin, highest creatinine, highest ALT will be registered in the eCRF blood work.

§ Overall survival from HIPEC registry is also acquired prior to interim analysis

## END OF STUDY

The end of study (EOS) is defined as 5 years after the last patient was included in the study, when long-secondary outcomes can be evaluated.

# SELECTION AND WITHDRAWAL OF SUBJECTS

## Subject inclusion criteria

1. Provision of written informed consent prior to any study specific procedures
2. ECOG Performance Status Score 0,1 or 2 alternatively Karnofsky 60-100
3. Adequate kidney, liver, bone marrow function according to laboratory tests
4. For females of childbearing potential, a negative pregnancy test must be documented
5. ≥ 18 years old and <75 years old.
6. Colorectal cancer with peritoneal metastases with or without liver metastases. The liver metastases have to have been removed prior to CRS+HIPEC or at the same time as the CRS+HIPEC. Maximum 3 simple liver metastases requiring non-complex liver resections.
7. All patients deemed eligible for CRS and HIPEC according to clinical routine

## Subject exclusion criteria

1. Previous severe toxicity/allergic reactions to systemic chemotherapy agents oxaliplatin or irinotecan or 5-FU
2. Unable to tolerate intensified HIPEC treatment due to comorbidity
3. Metastasis other than peritoneum or liver
4. Complex liver-perenchymal sparing surgery or hemihepatectomy procedures are to be excluded.
5. Previous CRS or HIPEC
6. Pregnant or lactating (nursing) women
7. Active infections requiring antibiotics
8. Active liver disease with positive serology for active hepatitis B, C, or known HIV
9. Concurrent administration of any cancer therapy other than planned study treatment within 4 weeks prior to and up to 4 weeks after study treatment
10. Incomplete cytoreduction defined as completeness of cytoreduction score 1-3
11. Histopathology of other origin than colorectal cancer

## Withdrawal of subjects

The time from randomization to administration of intraoperative chemotherapy treatment is very short, basically a few hours. Thus, subject withdrawal will in principle not occur after randomization and prior to treatment. However, withdrawal may occur due to intraoperative complications leading to no HIPEC treatment administration. As these patients will not be randomized, they will not be included in the trial other than as a part of the fallout between informed consent and randomization.

Naturally, patients may opt to withdraw for any reason from the trial. In that case, all data collected up to the point of withdrawal will be included in the trial. No further quality of life questionnaires will be sent out after withdrawal has been requested. All other follow-up will also be discontinued.

## Monitoring subject compliance

No monitoring of subject compliance is necessary as all treatment is administered during surgery within hours of randomization during which the patient is anesthetized.

## WOMEN OF CHILDBEARING POTENTIAL (WOCBP)

Males being included in the trial that have WOCBP partners are required to use a condom from the time of treatment and 90 days postoperatively. This period may be extended in the case of adjuvant therapy. For non-pregnant WOCBP partner, contraception is recommended.

WOCBP being included in the trial shall have a highly effective negative pregnancy test performed as part of the inclusion criteria. The following highly effective birth control (<1% failure rate) should be in use prior to study inclusion and continue for 6 months after surgery (extended another 6 months in the case of adjuvant therapy administration).

1: Combined (estrogen and progestogen containing) hormonal contraception associated with inhibition of ovulation

o oral

o intravaginal

o transdermal

2: Progestogen-only hormonal contraception associated with inhibition of ovulation

o oral

o injectable

o implantable

3: Intrauterine device (IUD)

4: Intrauterine hormone-releasing system ( IUS)

5: Bilateral tubal occlusion

6: Vasectomised partner

7: Sexual abstinence

# TREATMENT OF SUBJECTS

## Treatment administration

A previous dose-titration study from France has shown that 400 mg/m^2^ of both oxaliplatin and irinotecan HIPEC were the maximum tolerated dosages. However, after some further use, the doses have been around 360mg/m^2^ in clinical routine due, primarily, to neutropenia [11]. In this trial, this slightly lower dosage will be kept (even though neutropenia appears to be a positive factor for disease-free survival without risk of increase in other postoperative morbidity), in order to be able to have some margin upon which to dose-titrate the 5-FU 24-hour EPIC.

At the discretion of the surgeon (e.g. co-morbidity), the surgeon/oncologist is allowed to decide on a dose reduction; either give the full study dose – 100% of planned dosing, or 25% dose reduction – which is a 25% reduction of all chemotherapeutic treatments during the HIPEC procedure.

The HIPEC may be administered with any HIPEC administration device that is approved for this purpose. The administration may be done with the open or closed abdominal technique. The targeted temperature is 41-42 degrees Celsius. The chemotherapy perfusion time is 30 min and the perfusion rate is targeted to 1 liter per min, but may be adjusted as needed to keep the HIPEC treatment going and keep the temperature stabile in the abdomen. A minimum of 2 temperature probes are required in the abdomen to monitor the target temperature. Gastrointestinal reconstruction is optionally performed prior to or after the HIPEC procedure.

The EPIC procedure may commence once the abdomen is completely shut which includes completing skin closure and stoma suturing. Administration is described below in Arm B. At least two drains must be left in the abdomen with one in the upper abdomen and one in the pelvic region which are required in order to administer EPIC. The drains must be sutured to the skin and should not allow fluid leakage. A slightly diagonal trajectory through the abdominal wall with purse string sutures is an appropriate method of drain placement (other methods are allowed as long as no fluid leakage occurs).

Arm A (HIPEC) Standard treatment (normal dose and 1 dose level reduction)

- Oxaliplatin – 460 mg/m^2^ (HIPEC) – 25% dose reduction 350 mg/m^2^
- 5-FU – 400 mg/m^2^ IV bolus (Intraop) – 25% dose reduction 300 mg/m^2^
- Calcium folinate 60 mg/m^2^ IV (Intraop)
- Granulocyte colony stimulating factor given postoperative only as needed when the leukocyte count falls below normal range.

Arm B (HIPEC + EPIC) Experimental treatment (normal dose and 1 dose level reduction)

- Oxaliplatin – 360 mg/m^2^ (HIPEC) – 25% dose reduction 270 mg/m^2^
- Irinotecan – 360 mg/m^2^ (HIPEC) – 25% dose reduction 270 mg/m^2^
- 5-FU – 400 mg/m^2^ IV bolus (Intraop) - 25% dose reduction 300 mg/m^2^
- Calcium folinate – 60 mg/m^2^ IV (Intraop)
- 5-FU – 250-850 mg/m^2^ IP 24-hour dwell in 400ml of 9% saline solution (End of op) (25% dose reduction 190-640 mg/m^2^)
  - The EPIC treatment is only 1 day and is administered after the abdomen is completely sutured in the operating theatre. The dose will be divided equally into 2 injections á 200ml each through two abdominal drains. The drains may be flushed with 20ml saline solution so that the chemotherapy is not lingering in the drain catheter. Afterwards, all drains are clamped for 16 hours. They are then opened in order to drain the abdominal cavity postoperatively.
- Granulocyte colony stimulating factor (G-CSF – e.g. filgrastim 5µg/kg/day) administered prophylactically on postoperative day 4 to 8 (5 days). If the neutrophil count is under normal range after 5 days of prophylactic treatment, the treatment may continue until the count is normalized, as clinically indicated. If leucocytosis occurs, the prophylactic treatment can be terminated earlier than after 5 days.

G-CSF may be administered as needed before or after the prophylactic period if an early or late neutropenia is detected.

## Description of investigational products

Irinotecan – 360mg/m^2^: This investigational drug will be procured in a syringe by the hospital pharmacy for a one-time administration. It will be injected into the HIPEC perfusate circulating in the hyperthermic pump via the injection site. It will be injected together with oxaliplatin that is a non-investigational drug that all patients will receive irrespective of arm.

5-fluorouracil – Maximum tolerated dose of 1-day EPIC to be determined: This investigational drug dose will be divided into two separate syringes. It will be injected through two of the remaining passive abdominal drains. After injection, the drain will be cleared from chemotherapy with a saline solution injection. The drains will be clamped. The estimated peritoneal clearance is 8-10 hours. The clamps need to be closed 16 hours. They are then opened for drainage of fluid collections to occur according to our clinical routine for abdominal drains in HIPEC.

## Packaging and labelling of investigational products

5-Fluorouracil is found in the dose 50mg/ml in 20 ml or 100ml glass injection bottles that are packaged in a carton. Even though we advocate the use of Fluorouracil Accord, Fluorouracil Tevo may also be used. They have the exact same dosage, glass bottle sizes, and packaging type.

Irinotecan is provided by three companies in Sweden: Accord, Actavis, and Fresenius Kabi. All of the companies use the same dosage of 20mg/ml. The have different sizes of injection bottles packaged in cartons. All three companies may be used depending on what the local hospital pharmacy has in storage. Since there is no blinding in the study and the drugs are post-market drugs, the investigational drugs of irinotecan and 5-fluorouracil for intraperitoneal administration will be labelled as is with irinotecan and 5-FU and with respective doses on the syringes.

## Storage and handling

Both drugs are actively used every week in the medical oncology outpatient clinics in all four participating centers in routine oncology treatment. Thus, each center hospital has a constant turnover of the investigational medicinal product (IMPs) fluorouracil and irinotecan. Due to the common use of these drugs, there is little need for specific IMP drug oversight. Each pharmacy will store, handle, dilute and prepare the drugs according to the specifics as described in the published marketing drug IMP brochure (see FASS.se).

## RandomiZation and blinding

Patient randomization will be integrated into the HIPEC registry and will be performed intraoperatively after abdominal exploration and after a CC 0 result is deemed achievable. For patients with isolated peritoneal metastases, there will be center stratification and PCI stratification (PCI 1-10 vs 11+). For patients with combined liver and peritoneal metastases, there will be only PCI stratification (PCI 1-10 vs 11+). Multivariable regression will be used in the results section in order to account for skewed differences in baseline differences. See the planned statistical analyses (13.2).

There is no blinding involved in this trial. This is mainly due to the fact that one arm will need to receive G-CSF treatment postoperatively; and thus, the treating physician needs to know which arm the patient is allocated to. The primary endpoint is recurrence free survival which is determined by radiologists on a CT scan. The radiologists will not be privy to the treatment allocation. Thus, there is a blinding of primary endpoint evaluation.

## Concomitant therapy

All necessary medical treatment needed to manage possible surgical or HIPEC/EPIC related complications are permitted. Likewise, all medications that the patient is currently taking may be administered as usual unless a very specific drug interaction needs review by a site investigator. In which case, depending on the medication, it may be temporarily discontinued in the postoperative period. Concomitant medication administered the day before and the day of surgery is registered in an eCRF.

Adjuvant systemic chemotherapy is administered according to each center’s clinical routine and will be registered in the HIPEC registry.

The patient may not join another adjuvant treatment trial study prior to recurrence without the consent of the study committee. A judgement concerning the effect on this current study will need to be performed before allowing inclusion into a concomitant study, which by principle will generally not be allowed unless it is systematically applied to all included patients and a renewed sample size calculation has been performed.

## Accountability of investigational products

The study requires a less rigorous accountability as the use of both drugs is commonplace in routine oncological treatment of colorectal cancer. The drug is ordered from the operating room the same morning that it is administered. It is delivered approximately 1-2 hours after a prescription is sent to the pharmacy from the operating room. If the products are not administered as planned due to an unforeseen event during surgery, they will be returned to the pharmacy for destruction and logged as not administered. This will be noted in the registry treatment follow-up.

## rOLE AND RESPONSIBILITIES OF TREATING PHYSICIANs

The investigators and co-investigators include almost all HIPEC treating physicians in Sweden as this is a small subspecialty. Each site investigator and co-investigator is responsible for all eCRF data input and study related visits. GCP training is required of all investigators. GCP training can be offered to those needing the training. Financial costs for GCP training may come from the study budget, if needed. They may enlist the help of a GCP trained research nurse for study assistance. Each site may delegate responsibility of study visits to other physicians in the same department by a signed delegation ensuring GCP training as well.

# ASSESSMENT OF EFFICACY

The primary endpoint in the efficacy trial is RFS after 12 months. Data from the one-year postoperative clinical follow-up that is registered in the HIPEC registry will be retrieved. Besides the site of relapse, also the date of recurrence is retrieved. It is defined as the date of the radiological examination that identifies the recurrence. Crude overall survival will also be assessed at the end of the trial follow-up as a secondary endpoint for the trial. The HIPEC registry is automatically connected with the Swedish death registry and therefore the overall survival follow-up is completely automatic as to the date of death. However, cause of death will also be retrieved from the cause of death registry in order to perform exploratory analyses of cancer specific survival. Likewise, quality-of-life will be retrieved from the HIPEC registry as well.

## Clinical and laboratory assessments

### Timing and method of the assessment

The dose titration is assessed continuously as each dose level is completed. All morbidity data is retrieved from the HIPEC registry.

Patient follow-up:

Mandatory

- Abdominal and thoracic CT scan at baseline before surgery and 12 months postoperatively.
- Treatment administered (CRS+HIPEC+EPIC variables)
- Morbidity to be submitted within 30 days from treatment administration. SAE and SUSAR are to be reported immediately (a separate section will make this possible in the HIPEC registry)
- Overall survival follow-up at
- 5 years from the HIPEC registry

Clinical routine

- The following follow-up is standard in Sweden after CRS+HIPEC: CT scan and tumor markers every 6 months for 2 years and then annually for another 3 years. Since all these follow-ups are not included in the HIPEC registry, we will not include them in the schedule of investigational events.
- EORTC QLQ-C30, CR29, and STO22 questionnaires filled out at baseline, 3, 12 and 36 months after surgery using the established QoL database. All patients are included in the quality-of-life registry as part of the clinical quality assurance follow-up, unless they specifically have requested not to be included.

Optional: Sending intraoperative tumor tissue to Uppsala for chemoresistance testing within 48 hours. Tissue is discarded right after testing. No biobanking will occur.

#### CT scan

A relatively recent CT scan within 6 weeks of the treatment administration is needed. Likewise, a radiologist evaluation of the 12-month CT scan needs to be registered in the HIPEC registry. If a recurrence has occurred prior to 12 months, the earlier date for the CT scan where the recurrence was noted is to be used.

#### Tumour markers

Carcinoembryonic antigen (CEA), Cancer antigen 19-9 (CA 19-9), Cancer antigen 125 (CA 125).

#### Health-related quality-of-life assessment

EORTC QLQ-C30 and CR29 and STO22 forms will be used to evaluate quality-of-life [10]. These forms will be sent out at baseline, 3, 12, and 36 months. The primary outcome is the comparison of questions 29 and 30 from the QLQ-C30 at 12 months rated 1-7, where 1 is very poor and 7 is excellent.

QLQ-C30 questions for QoL assessment:

29. How would you rate your overall health during the past week?

30. How would you rate your overall quality of life during the past week?

#### Translational study

To assess chemotherapy resistance and their capacity to predict treatment outcome, tumor tissue will be sent during surgery at Uppsala University Hospital, Uppsala, Sweden, to the chemotherapy resistance testing lab at the hospital. This part of the study is optional for the other three HIPEC centres in Sweden. Using the IC_50_ value attained from the test results [11], a Cox proportional hazards model will be implemented to evaluate whether the resistant oxaliplatin/irinotecan/5-FU values (top 1/3 of the whole study cohort’s IC_50_ values vs bottom 2/3) lead to poor survival outcome, both overall survival and recurrence-free survival. Patients having received at least one sensitive drug during treatment will be compared to the patients having received only resistant drugs.

# ASSESSMENT OF SAFETY

## Timing and methods for Assessing safety parameters

In the dose titration study, all adverse events will be registered in the separate adverse event eCRF section of the HIPEC registry (a section only used for trial purposes) as soon as possible after the event has occurred. The responsible investigator will need to alert the Sponsor and the DMC chairperson if a serious adverse event has occurred within 24 hours. A decision by the chairman of the DMC is issued whether to paus the trial for DMC review. Non-serious adverse events need not paus the trial accrual. Formal assessment by the DMC will be performed within 30 days after the last patients’ surgery on every dose level before moving to the next dose level.

In the subsequent efficacy study, registering the morbidity can be done within 4 weeks of discharge from the hospital. It will be registered in the HIPEC registry. Formal safety review by the DMC will only be performed during the interim safety evaluation, lest unexpected serious adverse events occur requiring an earlier review (all site investigators may initiate an earlier morbidity review).

In case of SAE (with at least a possible relationship to the study medication) or SUSAR, the sponsor will be responsible for informing via email all four participating HIPEC centers via their respective site investigator and site co-investigator that a SAE or SUSAR has been reported. An evaluation by the sponsor and the responsible site investigator will accompany the report. All DMC reviews will be released as well via email in the same manner

.

## Definition, assessment and causality of adverse events

An AE is any untoward medical occurrence that does not necessarily have to have a causal relationship with the investigational product. An AE can be any unfavourable, unintended clinical sign, symptom, medical complaint or clinically relevant change in laboratory variables or clinical tests. Accidents, operations not pre-planned, changes in medication or deterioration in concurrent illness are also considered as AEs.

The Investigator should rate the intensity of any AE as follows:

*Mild:* Defined as Clavien-Dindo grade I and II.

*Moderate:* Defined as Clavien-Dindo grade 3a.

*Severe:* Defined as Clavien-Dindo grade 3b-5.

The Investigator shall judge whether or not, in his/her opinion, the AE is associated with the study treatment. When stating the causality, the following nomenclature should be used:

*Unrelated:* There is little or no chance that the study treatment caused the AE.

*Possibly related:* The association of the AE with the study treatment is unknown, however, the AE is not clearly due to another condition.

*Probably related:* A reasonable temporal association exists between the AE and the study treatment. Based on the Investigator’s clinical experience, the association of the AE with the study treatment seems likely.

## Definition of serious adverse events

A serious AE is any untoward medical occurrence that at any dose:

- Results in death.
- Is life-threatening at the time of the event.
- Requires inpatient hospitalisation.
- Requires prolongation of existing hospitalisation.
- Results in persistent or significant disability/incapacity.
- Is a congenital anomaly/birth defect.

## DATA MOnitoring committee

The DMC which will include three external clinical reviewers will be responsible for making decisions on whether to move to the next dose level, as well as making decision to continue the phase III trial efficacy study after the interim safety evaluation (see interim analysis in the statistics section). The work of the DMC will be described in a DMC charter.

## Safety parameters

Clavien-Dindo morbidity was chosen as the most relevant morbidity grading system. The oxaliplatin/irinotecan/5-FU combination for systemic chemotherapy is already well known in oncology under different acronyms (FOLFIRINOX, FOLFOXIRI). As such, the toxicity profile of this triple combination is already known. This study will assess whether it is compatible with a surgical intervention using the Clavien-Dindo surgical classification. However, some basic blood chemistry as specified in section 8.3 (extended part of the HIPEC registry employed only for the dose titration study) will be added to verify the rate of non-surgical toxicity. As the 24-hour EPIC has not previously been dose-titrated, we will titrate to a maximum of 850 mg/m^2^. Clavien-Dindo 3a was excluded as a stopping factor due to its common use postoperatively after CRS+HIPEC for draining fluid collections (most commonly pleural effusion often caused by diaphragmatic stripping of the peritoneum and is dependent upon the extent of the stripping).

All patients will be reviewed at each of the dose levels by the DMC prior to advancing to the next level. It is Clavien-Dindo grade 3b-5 morbidity that will be reviewed. A dose level with ≤1/3 or ≤2/6 with 3b-4b morbidity may proceed to the next dose level. Max tolerated 3b-4b morbidity is 33 %. Statistical testing is unnecessary. All grade 5, leading to death, will halt study progression. A formal review with the DMC will ensue before the study will be allowed to continue or be terminated permanently. Two grade 5 events may be cause for study termination after DMC review.

### Interim safety analysis of the Efficacy study

After randomization, the Clavien-Dindo grade 3a-4b morbidity is expected to be approximately 25-35% when treating patients with the dose of 5-FU determined from the dose titration study. During the interim analysis of the efficacy part of the trial, the DMC will use the morbidity rate of the standard arm and compare it to the experimental arm. If all Clavien-Dindo grade 3-4 complications resolve in-hospital and occur at a maximum rate of 40%, the trial will pass the interim review. If complications lead to permanent sequelae or the rate is higher than 40% a detailed review by the safety committee will ensue prior to letting the efficacy study continue. Comparison with the standard arm will assist in determining the risk/possible benefit ratio. In particular, if the mortality rate is increased, it will be grounds for discontinuing the trial. The trial may be allowed to continue despite increased morbidity if the mortality rates are the same and if the morbidity is of a temporary nature, not leading to permanent functional loss. The decision will be made by the DMC. If necessary, dose reduction may be considered in order to continue and complete the efficacy study.

## Reporting of adverse events, serious adverse events, AND suspected unexpected serious adverse reactions

All adverse events will be reported in a separate eCRF adverse event report section of the HIPEC registry created for this trial. It is the responsibility of the site investigator or co-investigator to fill out an eCRF Adverse Event form in the HIPEC registry. All investigators have access through their SITHS ID cards to the registry. One form per AE is to be filled out. It may be filled out as part of the postop follow-up at predetermined time points of follow-up, but it may be filled out in between these time points since the patients are admitted to the hospital and under daily surveillance during 10-14 days. If classified as SAE it needs to be filled out within 24 hrs from being informed about the event.

Serious adverse events with a possible or probable relationship to the study medication including events leading to a deadly outcome need to be brought to the attention of the DMC. The adverse event eCRF section of the HIPEC registry will include a list box if an adverse event needs to be brought to the DMC for review. An automated email will be sent to the coordinating investigator (who is also the sponsor representative) who will be responsible for discussing the event with the DMC.

The Investigator’s initial report of serious AEs should as far as possible be supplemented by detailed information on diagnosis/symptoms, the relationship with the start of treatment and any further relevant data in the eCRF section.

If the SAE is assessed as unexpected and possibly/probably related to the IMP, i.e. a Suspected, Unexpected Serious Adverse Reaction (SUSAR) that are fatal or life-threatening, it shall be reported to the Medical Products Agency (MPA) as soon as possible and at the latest within 7 calendar days from the first knowledge by the Sponsor. The report shall as far as possible be supplemented by additional information, but at the latest within a further 8 calendar days. It is the responsibility of the Sponsor to report SUSARs to the MPA. It will be reported on CIOMS forms by e-mail to be registered in the EudraVigilance database by MPA.

The reporting of other SUSARs to the MPA shall be done as soon as possible, but not later than 15 calendar days after the first knowledge by the sponsor.

## Follow-up of adverse events and serious adverse events

All patients are followed up within 3 months postoperatively within normal clinical routine to evaluate the potential long-term effects of previous adverse events. If an AE is not recovered after 3 months, it will be followed up until recovered or stabilized.

## ANNUAL Development safety update report (DSUR)

As long as the study is ongoing, the Sponsor will annually send a summary of all SAEs to the MPA. The report will also summarize the safety and the risk for the subjects in the trial. The report will contain an updated risk/benefit summary.

## Procedures in case of medical emergency

The Investigator should ensure that there are procedures and expertise available to cope with emergencies during the study.

If an emergency occurs, please notify the Sponsor contact person:

Name: Peter Cashin, MD, PhD

Telephone: +46 (0)18 6174304

E-mail: peter.cashin@surgsci.uu.se

# STATISTICS AND DATA MANAGEMENT

## Data management

The patient is registered preoperatively in the HIPEC registry during the preoperative visit at the surgical outpatient clinic. This is the time that informed consent is made. If the patient opts to join the trial, there is a check box for the phase I dose titration study, which will change to phase III efficacy study after phase I is complete in the HIPEC registry. Once included, the HIPEC registry will automatically set reminders to site investigators when data needs to be put into the registry. Clinical registries are very good sources for researchers, however, the timing of data input is sometimes late, thus automatized reminders will be used to keep the registry constantly up-to-date during this trial.

Data input will be reviewed by the monitors. Incomplete data, as found by the monitors, will be listed in a data clarification form to be sent to respective site to resolve inconsistencies and missing information. A copy will be returned to the sponsor.

The HIPEC registry includes a number of variables that are not considered important for monitoring. The following variables are defined as monitoring points:

1. Study registration and diagnosis

(the study registration visit charting – study ID number, inclusion criteria, and diagnosis)

1. HIPEC/EPIC Treatment administered
2. Adverse events
3. Blood chemistry toxicity and medications
4. 12-month RFS as defined by the radiologist report

As part of informed consent, the patients will allow monitoring through the NPÖ (Nationella Patient Översikten) so that access to source data is made possible. The medical charting through the NPÖ is defined as the source data for all monitored variables.

## Statistical analysis

### Statistical methods

#### Dose titration study

The dose titration study will include 3 patients at each dose level, with a possibility to include 3 more patients if more than 1 patient experiences morbidity grade 3b-5 (3 + 3 design). In order to complete all 5 dose levels 15-30 patients will be needed depending on how often the second three patients are needed at each dose level. The toxicity follow-up is 30 days if no incidents occur. If the postoperative rehabilitation is slower or an incident occurs, the follow-up period may be extended by the site Principal Investigator or Co-investigator. Circa 3-4 colorectal cancer patients are treated with CRS+HIPEC each week in Sweden. Dose titration can be completed within 15-30 weeks.

#### Interim analysis

The Clavien-Dindo grade 3-4 morbidity is expected to be approximately 0.35 (35%) for patients receiving the maximum dose of 5-FU. Allowing for a small increase in morbidity to 0.40 and an accepted 95% confidence interval width of 0.15, at least 66 patients are needed in the experimental arm to determine whether the arm deviates from the expected morbidity rate. The total sample size will be 140 (70 per arm) for an interim trial safety evaluation (circa 50% of trial included). If the Clavien-Dindo grade 3-4 morbidity falls outside of the 40% mark, then a Fisher’s exact test comparison between the standard and experimental arm will be performed together with a mortality comparison. Concerning in-hospital mortality, a maximum rate of 5% will be set. This planned interim safety evaluation will be unpublished. If the morbidity is increased beyond the set mortality limit or the increase is of a more serious nature, the trial will either be discontinued or alternatively, depending on the character of the complications, a lower dose level may be employed.

The overall survival and recurrence free survival will be reviewed during this interim safety evaluation to see if a primary endpoint of overall survival is attainable with increased cohort size instead of 12-month RFS. If attainable depending on inclusion rate, the endpoint will be changed, and a new sample size calculation will be performed. Likewise, a choice between 3 or 5 year follow-up will be made. Alternatively, trial futility will be determined if no differences at all are forthcoming even in the RFS setting. In this case, the trial will close prematurely. A decision to close the trial is made by the sponsor in conjunction with site investigators.

Possible changes of dose level, endpoint, sample size calculation or other substantial changes will be submitted to the MPA and ERA as an amendment to the protocol.

#### Main efficacy study **(Changes have been made to analysis plan – please refer to appendix for the updated analysis plan)**

##### Primary outcome analyses of 12-month RFS

- ~~The primary analysis for RFS efficacy will be a multivariable logistical regression analysis with 12-month RFS as endpoint including the following 7 parameters: treatment arm, age, PCI, use of systemic perioperative chemotherapy (neoadjuvant and/or adjuvant), liver metastases resection, colon or rectal primary, and lymph-node metastasized primary tumor.~~
- ~~A Fisher’s exact test for 12-month RFS between the arms~~

~~Secondary outcome analyses of total RFS~~

- ~~A Kaplan-Meier curve with two-tailed log rank test between the arms with RFS up to 5 years as endpoint (i.e. time to recurrence)~~
- ~~A multivariable Cox regression analysis with RFS (i.e. time to recurrence) as endpoint including the following 7 parameters: Treatment arm, age, PCI, use of systemic perioperative chemotherapy (neoadjuvant and/or adjuvant), liver metastases resection, colon or rectal primary, and lymph-node metastasized primary tumor.~~
- The same analyses above may be run against peritoneal recurrence-free survival.

If any of the analyses above show a significant result, the trial will conclude that the intensified HIPEC arm indeed improves the locoregional effect of HIPEC compared to the current standard HIPEC.

If the interim analysis results demonstrate that overall survival is attainable as primary endpoint, a new statistical analysis will be set up after a renewed sample size calculation.

##### Secondary outcome analyses of overall survival and QoL:

- Overall survival up to 5 years will be compared with Kaplan-Meier and log-rank test as well as multivariable Cox regression using the following same variables as RFS: Treatment arm, age, PCI, use of systemic perioperative chemotherapy (neoadjuvant and/or adjuvant), liver metastases resection, colon or rectal primary, and lymph-node metastasized primary tumor.
- Quality of life between the two arms will be compared. The primary outcome is the comparison of questions 29 and 30 from the QLQ-C30 at 12 months and 36 months. The primary statistic will be a Mann-Whitney U test of difference for both question 29 and 30 between the arms. The secondary statistic will be a comparison of mean differences for question 29 and 30 between baseline and 12-months using a Freidman’s repeated measures ANOVA test. Specifically, differences between the sexes will be investigated.
- Three specific subgroup multivariable analyses using RFS as endpoint will be performed using the same variables as above for correction: (1) PCI score – the score will be grouped 1-10, 11+, (2) Signet cell cancer, (3) previously given oxaliplatin-based therapy, (4) +/- livermetastases.
- Further exploratory and post hoc analysis of subgroups will be performed: multivariable analysis for overall survival and forest plots.

## Determination of sample size

Dose titration study

15-30 patients depending on how many dose levels require 6 patients.

Interim trial safety evaluation

140 patients (70 in each arm) which is close to 50% of the sample size for an extended safety analysis. See 13.2.1.2.

Efficacy study

Using an alpha of 5% and beta of 80% and a 12-month recurrence free survival benefit from 50% till 66% from a recent retrospective study comparing Ox HIPEC vs Oxiri HIPEC for colorectal cancer (figure 1, [6]), 148 patients is needed in each arm. With 10% loss to follow up or violation rate, 163 patients in each arm will be needed. In total, 326 patients are to be included in the colorectal cancer group which will define the sample size.

In total, approximately 341-356 patients will be recruited for the phase I/III trial which will be included from 4 centers with the following estimations:

Uppsala – 114-118 patients

Stockholm – 114-118 patients

Malmö – 57-59 patients

Göteborg – 57-59 patients

Aarhus – patient count pending

Oslo – patient count pending

The CRS and HIPEC operation rate in Sweden is approximately 180 patients/year. Two-thirds are diagnosed with colorectal cancer leaving 120 potential candidates to be included in the study. If we manage a 90% inclusion rate, the dose titration part of the trial will take 6 months followed by another 4.5 years to study efficacy (see 20.5 for further details). The trial program will take approximately 5 years to complete.

##

## Statistical significance

The statistical significance level will be set at *p*=0.05.

## Criteria for the termination of the trial

If no numerically and clinically relevant differences are found in either RFS, overall survival, peritoneal RFS, or pattern of recurrence at 12 months or during the whole study follow-up, then the study can be deemed futile and may be closed prematurely at the time of the interim analysis.

## Missing data management

If missing data is identified during study data acquisition, then it may be pertinent to consult hospital charting to rectify missing data. However, if the data is not available and cannot be acquired, then a multiple imputation analysis may be performed.

## Intention-to-treat analysis

The trial will be intention-to-treat. Thus, all patients being randomized are included in the trial. Since treatment is administered within hours of randomization, there will likely be no or minimal fallout of subjects between randomization and treatment administration.

# Direct access to source data/documents

The Investigators/institutions will permit study-related monitoring, audits, review and regulatory inspections, providing access to source data/hospital records. Sponsor verifies that each patient has consented in writing to direct access to the original source data/hospital records by the use of written patient information and signed Informed Consent, which also includes remote access via NPÖ (Nationella Patient Översikten).

During the monitoring, the data recorded in the HIPEC registry (and eCRF section) will be controlled for consistency with the source data/hospital records by the study monitor (source data verification). Any discrepancies of data will be documented and explained in the monitoring reports. The data that is to be monitored has been specified in the section 13.1 “Data Management”.

As the sponsor representative and coordinating investigator is the same person, access to source data is available also for the sponsor. All site investigators are required to register the data in the HIPEC registry as determined by this protocol. If problems with registering data occur, the study monitor may do a site visit in order to acquire the necessary data for completing the required analyses.

# Quality control and quality assurance

This trial program will employ a regional clinical trial unit (UCR - Uppsala Clinical Research Center) with many years of experience in running and setting up randomized clinical trials as well as registry-based randomized trials. Preliminarily, UCR will conduct the trial monitoring as well as support all necessary documentation and procedures needed to pass Swedish regulatory requirements.

## Source data

The following minimum amount of information should be recorded in the hospital records (the relevant hospital charting which includes this information is specified in the section 13.1):

- Clinical study number. (Preoperative visit)
- Subject identification. (Preoperative visit)
- Date when patient information was given and when signed Informed Consent was obtained. (Preoperative visit)
- Diagnosis. (Preoperative visit)
- Fulfillment of inclusion criteria. (Preoperative visit)
- Specification of visit dates, concomitant medication and any AEs. (Medication and blood chemistry results)
- Specification of study treatment and doses given. (Operating notes)
- Specification of the subject’s cessation in the study (e.g. withdrawal from follow-up).

Since the trial is registry based, the follow-up does not require separate eCRF evaluations. These evaluations can be automatically retrieved from the registry – both recurrence data, quality of life, and morbidity data.

## Monitoring

In accordance with the principles of Good Clinical Practice (GCP), monitoring of the study will be arranged by the Sponsor. During the study, the Monitor will have regular contacts with the study site(s), including visits to ensure that the study is conducted and documented properly in compliance with the protocol, GCP and applicable regulatory requirements. The HIPEC registry and quality-of-life registry will be monitored for consistency. The monitor work will be described in a monitoring plan.

The study sites may also be subject to quality assurance audit by the Sponsor as well as inspection by the MPA. The site Principal Investigator and other responsible personnel must be available during monitoring visits, audits and inspections and should devote sufficient time to these processes.

All study personnel should provide a curriculum vitae (CV) or equivalent documentation of suitability for their responsibilities in the study. All Investigators and other responsible personnel should be listed together with their function in the study on the signature and delegation list.

# ETHICS

## Ethical review authority

It is the responsibility of the Sponsor to obtain approval of the study protocol/protocol amendments, the patient information and the Informed Consent from the ERA before enrolment of any subject into the study.

## Ethical Conduct of the study

The study will be conducted in accordance with the protocol, applicable regulatory requirements, GCP and the ethical principles of the Declaration of Helsinki as adopted by the 18^th^ World Medical Assembly in Helsinki, Finland, in 1964 and subsequent versions (Appendix 21.5).

The main ethical considerations in this research project are related to the balance of procedural morbidity/mortality and efficacy/benefit. CRS and HIPEC treatment is a morbid procedure in itself, thus it is important to monitor that the morbidity does not lead to increased postoperative mortality. Furthermore, it is important that morbidity does not lead to increased long-term functional handicap. Most morbidity involved in this procedure is temporary. Out of 9 previous studies on EPIC, 3 studies indicated that there may be increased morbidity (but none for mortality) [7]. The other 6 studies showed no difference. The largest study [6] (group E is comparable to the intensified HIPEC arm in this protocol) shows no morbidity difference, no mortality difference, and no increased reoperation rate either. As such, it is not anticipated that this study will lead to a general increase in morbidity or mortality. However, this intensified HIPEC arm has not been followed up in a prospective systematic way. Neither has the maximum tolerated dose for a 24-hour 5-FU been determined. Therefore, this trial will begin as a phase I study that moves into a randomized open-label phase III study. During an interim analysis after half the patients in phase III part have been included, the morbidity results will be reviewed by the DMC before finishing the phase III step.

One specific morbidity that has been raised concerning intensified HIPEC treatment is neutropenia [11]. This is a specific morbidity that most likely will be increased with the intensified treatment. However, a recent study [8] investigating the specific effects of neutropenia showed that other serious side-effects were not increased nor mortality. Unexpectedly, neutropenia was a positive prognostic factor. Moreover, the effects of neutropenia are temporary and mitigated relatively easily with granulocyte-colony stimulating factor. Lastly, to monitor the patient’s perspective on the side-effect/benefit balance, a quality-of-life part of the trial will be included in order to follow-up health-related quality-of-life.

## Risk - benefits

As seen in the safety review from an earlier study [7] in Table 2, the morbidity of adding EPIC treatment to HIPEC is inconclusive. A few studies have shown increased morbidity while others have not. Due to the logistical problem of chemotherapy administration on the surgical wards and the possible morbidity increase, the EPIC treatment will be decreased from the standard 5 days to a one-time administration at the end of surgery. This will alleviate any potential increased risks with adding EPIC treatment to HIPEC.

**Table 4. Table 2 from Systematic Review [7]:**

Studies comparing HIPEC to the combination of HIPEC + EPIC (for 5 days) for lower gastrointestinal tumors with peritoneal metastasis following cytoreductive surgery.

| **Author, year, country** | **Origin** | **n** | **Treatment regimen** | **Grade 3 + morbidity** | **Survival analysis** |
| --- | --- | --- | --- | --- | --- |
| Glehen International | Colorectal | 271 112 | HIPEC HIPEC + EPIC | EPIC=more fistulasRR 1.7. p=0.032 | HIPEC + EPIC Better than HIPEC and EPIC alone but NS p=0.61 |
| Saxena Australia | Colorectal | 12 34 | HIPEC HIPEC + EPIC | 50% NS 30% | Not reported |
| Chua international | PMP | 1382 668 | HIPEC HIPEC + EPIC | No difference | HIPEC found to be an independent factor of better OS but not EPIC |
| Chua Australia | Colorectal Subgroup^a^ | 30 45 | HIPEC HIPEC + EPIC | 13% 16% | 19 months RFS. 19 months OS 33 months RFS. 38 months OS p=0.046. p=0.38 |
| Lam [12] Canada | Colorectal + High grade appendix | 37 56 | HIPEC HIPEC + EPIC | 19.6% p=0.01 43.2% | 6% 3Y RFS 46% 3Y OS. 21% 3Y RFS 50% 3Y OS. NS. NS |
| Sparks Australia | Appendix | 13 17 | HIPEC HIPEC + EPIC | Trend toward more complications with EPIC group NS | No difference |
| Tan Singapore | multiple | 69 42 | HIPEC HIPEC + EPIC | 25% p=0.048 58% | Not reported |
| Huang Australia | LAMN | 74 176 | HIPEC HIPEC + EPIC | 44.6% 48.3% | 64.5% 5Y OS. p=0.001 93.0% 5Y OS |
| Huang [13] Australia | PMCA | 118 67 | HIPEC + EPIC | 47.9% 53.7% | 30.5% 5Y OS. p=0.003 62.3% 5Y OS |

^a^Appendiceal neoplasms reported in later case control studies of the same unit.

HIPEC, Hyperthermic intraperitoneal chemotherapy; EPIC, Early postoperative intraperitoneal chemotherapy; NS, Non statistically significant; PMP, pseudomyxoma peritonii; OS Overall survival; RFS, Recurrence free survival; LAMN, Low-grade appendiceal mucinous neoplasm; PMCA, Peritoneal mucinous carcinomatosis of the Appendix.

An international collaboration on colorectal cancer with peritoneal metastases has demonstrated in manuscript format [6] that there is no increased morbidity connected with intensifying the HIPEC treatment both in terms of dual HIPEC drugs (oxaliplatin and irinotecan HIPEC) and in terms of adding EPIC (1-5 days of 5-FU) to the dual drug HIPEC treatment. Most importantly, there is no increased risk of mortality [6].

One morbidity that has been discussed separately is neutropenia. In a previous study conducted in France, the main increase in morbidity between oxaliplatin HIPEC and oxaliplatin/irinotecan HIPEC was neutropenia [11]. However, the risk of neutropenia was alleviated with G-CSF and it did not lead to increased mortality nor to an increase in other grade 3-4 adverse events. A new study has investigated the potential harm of postoperative neutropenia after HIPEC and not only did it not lead to increased other Clavien-Dindo grade 3-5 morbidity, but it was also a positive prognostic factor for treatment outcome [8].

As seen in Figure 1 in the beginning of the protocol, the survival benefit is significant between Single drug oxaliplatin HIPEC vs double drug HIPEC+EPIC. The estimated benefit gives a hazard ratio of 0.81, *p*=0.022.

The intensified arm (oxiri HIPEC + 5-FU EPIC) has been in use already within routine clinical practice primarily from one French institution that has treated 96 patients[6]. However, the use of single oxaliplatin HIPEC + 5 days of 5-FU EPIC has been in widespread use in many institutions, including Swedish clinics [5]. Our modified arm has only 1 day of EPIC instead of 5. Thus, the expected morbidity should be even less, and it is the view of the Study Committee that the potential benefits in a significant way outweigh the potential increased risk. However, in order to verify this systematically, the trial will have a two-step design – first a phase I dose-titration study to find the best 24-hr EPIC dosage of 5-FU; thereafter, follows a phase III open-label randomized trial.

An assessment of conducting this trial during the COVID-19 outbreak has been performed. All patients included in this trial will already be going for surgery and will receive a HIPEC treatment regardless of the current outbreak. This treatment is considered imperative. The only identifiable difference to date is the postoperative neutropenia which separates the experimental arm from the standard arm. However, due to the extensive surgery, all patients will be continuously monitored in-hospital until the postoperative neutropenic period is over. The anticipated start date for this trial will be after all hospital personnel in Sweden have received COVID-19 vaccinations. The risk/benefit ratio is therefore found to be in favor of trial continuation. One adjustment has been made per suggestion from the “GUIDANCE ON THE MANAGEMENT OF CLINICAL TRIALS DURING THE COVID-19 (CORONAVIRUS) PANDEMIC”. In order to decrease the need for travel, the 90-day follow-up may be performed via telephone or videocall unless there is an adverse event needing physical examination.

## Patient information and informed consent

It is the responsibility of the Investigator to provide each subject with full and adequate verbal and written information about the objectives, procedures and possible risks and benefits of the study. All subjects will be given the opportunity to ask questions about the study and will be given sufficient time to decide whether or not to participate in the study. The written patient information must not be changed without prior discussion with the Sponsor.

The subjects will be notified of their voluntary participation and of their freedom to withdraw from the study at any time and without giving any particular reason. Subjects must also be informed that withdrawing from the study will not affect their future medical care, treatment or benefits to which the subject is otherwise entitled.

The Investigator is responsible for obtaining written Informed Consent from all subjects (or their legally acceptable representatives and/or witnesses, where applicable) prior to enrolment in the study.

The subjects will consent to:

- Participating in the study.
- Personnel concerned at the manufacturer and regulatory authorities to gain full access to hospital records, to control the data collected in the study in NPÖ.
- Recording, collection and processing of data and storing of data in a database.
- Possible transfer of information from the study to countries outside the European Union (EU).
- Possible storing of study samples in a biobank.

It should be clearly stated that the data will not identify any subject taking part in the study, in accordance with the EU General Data Protection Regulation (GDPR).

A copy of the patient information and the Informed Consent form will be given to the subject. The Investigator (or a designated co-Investigator) who gave the verbal and written information to the subject will sign the Informed Consent form. The Investigator will file the signed Informed Consent forms in the Investigator’s File.

It is suitable to notify the subject’s family doctor of the subject’s consent to participate in the study.

# Data handling and record keeping

## Case Report Forms

A CRF is required and should be completed for each included subject. The subject’s identity must always remain confidential. All information in the CRFs should be in English.

The CRFs will be an integrated part of the HIPEC registry. Data clarification forms that lead to changes in the HIPEC registry will be tracked in the registry and as well as who makes them.

## Record keeping

To enable audits and evaluations by the Sponsor and inspections by regulatory authorities, the Investigator will keep records (essential documents) of the study for at least 10 years (or as required by current regulations). This includes any original source data related to the study, the subject identification list (with subject numbers, full names and addresses), the original signed Informed Consent forms, copies of all study data and detailed records of investigational products disposition.

The phase I/III trial will mainly use registry-based data. A few variables related to the Swedish Medical Products Agency requirements, and patient consent forms and randomization variables will be added to the HIPEC registry as a separate digital CRF under a separate ongoing study section (as such all CRFs will be integrated into the HIPEC registry). All variables in the HIPEC registry will be extracted. Furthermore, at the conclusion of the trials, data from the national Swedish colorectal cancer registry will be extracted for each patient in order to retrieve relevant clinic-pathological variables related to the primary tumor. All handling of data will be according to the Swedish Data Inspection regulations and the European GDPR legislation.

# INSURANCE

All patients treated in Sweden are insured by the Swedish Patient Insurance (Landstingens Ömsesidiga Försäkringsbolag) and the Swedish Drug Insurance (Läkemedelsförsäkringen). Each respective international site will have supplemental appendix outlining insurance policies for each respective country.

# PUBLICATION POLICY

All information not previously published concerning the investigational product, including patent applications, manufacturing processes, basic scientific data, etc., is considered as confidential and will remain the sole property of the Sponsor. The Investigator agrees to use this information only in connection with this study and will not use it for other purposes without written permission from the Sponsor.

After completion of the study, the statistical analyses will be performed by the Sponsor and the results will be presented to the Investigators. Based on these data, the Sponsor, in cooperation with the Investigators, will prepare a clinical study report. The report will be submitted to the MPA and may form the basis for a manuscript intended for publication in a medical/scientific journal. Each phase of the EFFIPEC clinical trial program will be published separately with two primary planned articles according to the primary endpoint of each trial phase.

# SUPPLEMENTS

## Changes of the study protocol

No change in the study procedures will be effected without the mutual agreement of the Investigator and the Sponsor (except where necessary to eliminate an immediate hazard to subjects). All changes of the final study protocol must be documented by signed protocol amendments. If substantial changes to the design of the study are made, the MPA and the IEC will be notified for review and approval.

## Application to regulatory authorities

Prior to initiating the clinical study, the Sponsor will submit an application for authorisation to conduct the study, including all required documents, to the MPA and ERA.

## Staff information

It is the responsibility of the Coordinating Investigator to ensure that all personnel involved in the study are fully informed of all relevant aspects of the study, including detailed knowledge of and training in all procedures to be followed.

## Criteria for termination of the study

The Sponsor reserves the right to discontinue the study prior to inclusion of the intended number of subjects but intends to exercise this right only for valid scientific or administrative reasons. After such a decision, all delivered unused investigational products and other study-related materials must be collected without delay and all CRFs must be completed as far as possible.

The study can be prematurely discontinued in the following cases (examples):

- Unexpectedly high proportion of adverse drug reactions.
- New findings about the investigational product(s) that changes the benefit/risk ratio.
- Study protocol is difficult to cope with.
- Recruitment of eligible subjects is far too low.
- Problems with manufacturing or stability of the investigational product(s).
- Unacceptable low Investigator, Sponsor or subject compliance.
- Critical change in personnel, administrative or scientific standards at the Sponsor or at the study center.
- No significant result will be obtained as anticipated.

## Study timetable

Dose titration study

Approximately 3-4 colorectal cancer patients are treated with CRS+HIPEC each week in Sweden. Dose titration can be completed within 15-30 weeks. At the discretion of the study committee, the sample size may be expanded upon depending on the type of morbidity encountered.

Efficacy study

In Sweden, approximately 120 patients with colorectal cancer and peritoneal metastases are treated annually. The inclusion and exclusion criteria are minimalized in order to allow inclusion of basically all patients accepted for surgery. Considering that the treatment options do not differ, in a practical aspect, for the patient (all treatments are conducted at the same time during surgery), we believe that the inclusion rate will be high. We aim to include 100 patients per year through the four HIPEC centers in Sweden (35 Uppsala, 35 Stockholm, 15 Malmö, 15 Göteborg). This would entail completing the inclusion within 3.5 years and finishing the main efficacy analysis after another 12 months (in total 4.5 years for the study). In total, the whole program is estimated to take 5 years.

# REFERENCES

1. Quenet F, Elias D, Roca L, et al. A UNICANCER phase III trial of hyperthermic intra-peritoneal chemotherapy (HIPEC) for colorectal peritoneal carcinomatosis (PC): PRODIGE 7. Eur J Surg Oncol. 2019;45(2):e17
2. Cashin PH, Sugarbaker PH. Hyperthermic intraperitoneal chemotherapy (HIPEC) for colorectal and appendiceal peritoneal metastases: lessons learned from PRODIGE 7. J Gastrointest Oncol. 2020. Accepted.
3. van Driel WJ, Koole SN, Sikorska K, et al. Hyperthermic Intraperitoneal Chemotherapy in Ovarian Cancer. N Engl J Med. 2018;378(3):230-240.
4. Yang XJ, Huang CQ, Suo T, et al. Cytoreductive surgery and hyperthermic intraperitoneal chemotherapy improves survival of patients with peritoneal carcinomatosis from gastric cancer: final results of a phase III randomized clinical trial. Ann Surg Oncol. 2011;18(6):1575-1581.
5. Fruhling P, Ghanipour L, Dranichnikov P, Enblad M, Birgisson H, Cashin PH. Oxaliplatin-based Hyperthermic Intraperitoneal Chemotherapy with Single Drug Versus Multiple Drug Treatment for Colorectal Cancer with Peritoneal Metastases: An Observational Cohort Study. Accepted J Gastroint Oncol. Jan 2021.
6. Oliver M. Fisher, Chris Brown, Jesus Esquivel, Stein G. Larsen, Winston Liauw, Nayef A. Alzahrani, David L. Morris, Vahan Kepenekian, Isabelle Sourrouille, Frédéric Dumont, Jean-Jacques Tuech, Cécilia Ceribelli, Béranger Doussot, Olivia Sgarbura, Mohammed Alhosni, Francois Quenet, Olivier Glehen, Peter H. Cashin. Hyperthermic Intraperitoneal Chemotherapy in Colorectal Cancer. Submitted for review.
7. Soucisse ML, Liauw W, Hicks G, Morris DL. Early postoperative intraperitoneal chemotherapy for lower gastrointestinal neoplasms with peritoneal metastasis: a systematic review and critical analysis. Pleura Peritoneum. 2019;4(3):20190007
8. Cashin PH, Ghanipour L, Enblad M, Morris DL. Neutropenia in colorectal cancer treated with oxaliplatin-based hyperthermic intraperitoneal chemotherapy: An observational cohort study. World J Gastrointest Oncol. 2020;12(5): 549–558.
9. EORTC Quality of life group. <https://qol.eortc>.
10. Cashin PH, Mahteme H, Graf W, et al. Activity ex vivo of cytotoxic drugs in patient samples of peritoneal carcinomatosis with special focus on colorectal cancer. BMC Cancer. 2013;13:435.
11. Quenet F, Goéré D, Mehta SS, Roca L, Dumont F, Hessissen M, Saint-Aubert B, Elias D. Results of two bi-institutional prospective studies using intraperitoneal oxaliplatin with or without irinotecan during HIPEC after cytoreductive surgery for colorectal carcinomatosis. Ann Surg. 2011;254(2):294-301.
12. Lam JY, McConnell YJ, Rivard JD, Temple WJ, Mack LA. Hyperthermic intraperitoneal chemotherapy + early postoperative intraperitoneal chemotherapy versus hyperthermic intraperitoneal chemotherapy alone: assessment of survival outcomes for colorectal and high-grade appendiceal peritoneal carcinomatosis. Am J Surg 2015;210:424–30.
13. Huang Y, Alzahrani NA, Liauw W, Soudy H, Alzahrani AM, Morris DL. Early postoperative intraperitoneal chemotherapy is associated with survival benefit for appendiceal adenocarcinoma with peritoneal dissemination. Eur J Surg Oncol. 2017;43(12):2292-2298.
14. Elias D, Raynard B, Bonnay M, Pocard M. Heated intra-operative intraperitoneal oxaliplatin alone and in combination with intraperitoneal irinotecan: Pharmacologic studies. Eur J Surg Oncol. 2006 Aug;32(6):607-13.

# signed agreement of the study protocol

To be signed per study centre.

**Title of the study:** “EFFIPEC – Efficacy of Hyperthermic Intraperitoneal Chemotherapy. Single-arm Phase I study, followed by an open-label, randomized, controlled registry-based phase III trial”

I, the undersigned, have read and understand the protocol specified above and agree on the contents. The study protocol, the Clinical Study Agreement and the additional information given about the investigational medical product will serve as a basis for co-operation in this study.

I agree to conduct the study according to this protocol and according to the ethical principles that have their origin in the Declaration of Helsinki and that are consistent with Good Clinical Practice and the applicable national laws and regulations.

**Principal Investigator**

| **Principal Investigator** | **Name, title**  **Address** |
| --- | --- |
| **Signature and date:** | **_______________________________________** |

# Appendices

## Declaration of Helsinki
