# Supplementary material for: Efficacy of hyperthermic intraperitoneal chemotherapy in colorectal cancer: A phase I and III open label randomized controlled registry-based clinical trial protocol
Source: PLoS One. 2024 Mar 4;19(3):e0294018. doi: 10.1371/journal.pone.0294018 (PMC10911585; doi:10.1371/journal.pone.0294018)
Supplement: S2 File — (DOCX) [file pone.0294018.s002.docx]

# Appendix – EFFIPEC v3

List of note-to-file changes in version 3 of the EFFIPEC trial protocol

1. Changes to Statistical Analysis Plan
   1. The method of randomization was not specified in the protocol. Block randomization was chosen. Patients with no liver metastases were randomized with center stratification and PCI stratification (1-10 vs 11+) with a block size of 6. Patients with liver metastases were randomized nationally with stratification of only PCI (1-10 vs 11+) also with a block size of 6.
   2. Due to liver inclusion in the block randomization. This variable will be removed from the adjusted multivariable analysis in the statistics section.
   3. Due to adjuvant therapy being administered after the randomization and treatment and, thus, the need for a time-dependent analyses, this variable will be removed from the adjusted multivariable analyses in the statistics section
   4. A switch in primary analysis was made from a multivariable logistic regression analysis to a multivariable Cox regression analysis of recurrence free survival. Primary analysis may be performed after a minimum of 12 months observation time. The remaining statistical analyses have been more clearly denoted as secondary analyses.
      1. “The primary analysis of efficacy for the phase III part of the trial will be a multivariable Cox regression analysis with RFS (i.e. time to recurrence) as endpoint including the following 6 parameters: treatment arm, age, PCI, use of systemic neoadjuvant chemotherapy, colon or rectal primary, and lymph-node metastasized primary tumor. The primary analysis can commence once all patients have been observed for a minimum of 12 months. Further secondary analysis will include a multivariable logistical regression analysis with 12-month RFS as endpoint including the following 6 parameters: treatment arm, age, PCI, use of systemic neoadjuvant chemotherapy, colon or rectal primary, and lymph-node metastasized primary tumor; a Fisher’s exact test for 12-month RFS between the arms; a Kaplan-Meier curve with two-tailed log rank test between the arms with RFS up to 5 years as endpoint (i.e. time to recurrence). The same analyses above may be run against peritoneal recurrence-free survival as secondary analyses as well.”
