# Supplementary material for: Efficacy of hyperthermic intraperitoneal chemotherapy in colorectal cancer: A phase I and III open label randomized controlled registry-based clinical trial protocol
Source: PLoS One. 2024 Mar 4;19(3):e0294018. doi: 10.1371/journal.pone.0294018 (PMC10911585; doi:10.1371/journal.pone.0294018)
Supplement: S3 File — (DOCX) [file pone.0294018.s003.docx]

Figure 1 – SPIRIT schedule

|  | STUDY PERIOD | | | | | | | | |
| --- | --- | --- | --- | --- | --- | --- | --- | --- | --- |
|  | Enrolment | Allocation | Post-allocation | | | | | | Close out |
| Time points | -1m | 0 d | 5 d ± 2 | 10 d ± 3 | 30 d +2m | 90 d  ±4m | 1y ±3m | 3 y ±3m | 5 y ±3m |
| **Enrolment** |  |  |  |  |  |  |  |  |  |
| eCRF Inclusion/screening | x |  |  |  |  |  |  |  |  |
| Signed informed consent | x |  |  |  |  |  |  |  |  |
| Randomization* / Allocation |  | x* |  |  |  |  |  |  |  |
| **Interventions** |  |  |  |  |  |  |  |  |  |
| CRS + HIPEC/EPIC treatment data  HIPEC registry treatment section |  |  |  |  |  | x |  |  |  |
| **Assessments** |  |  |  |  |  |  |  |  |  |
| Physical examination  (eCRF follow-up) | x |  | x | x |  |  |  |  |  |
| Telephone follow-up  (eCRF follow-up) |  |  |  |  | x | x | x | x |  |
| eCRF Drugs – medication/chemotherapy** | x |  |  |  |  | x |  |  |  |
| CT scan |  |  |  |  |  |  | x |  |  |
| eCRF Blood work*** |  |  |  |  | x |  |  |  |  |
| Questionnaire (QLQ-C30, CR29, STO22) HIPEC registry acquired | x |  |  |  |  | x | x | x |  |
| Morbidity from HIPEC registry (incl. eCRF AER/eCRF follow-up) |  |  | x | x | x |  |  |  |  |
| Overall survival HIPEC registry§ |  |  |  |  |  |  |  |  | x |
| Recurrence free survival HIPEC registry |  |  |  |  |  |  | x | x | x |

* only after the maximum tolerated dose of 5-FU has been determined

** (-1m) – previous chemotherapy and current medications, (90d) adjuvant chemotherapy administered

*** CEA, CA 19-9, CA 125 are taken preoperatively (-1m). Haemoglobin, C-reactive protein (CRP), white blood cell count (WBC), neutrophil count, platelet count, albumin, creatinine, alanine transaminase (ALT) are taken preoperatively and postoperatively 5 times during the first 10 days. The lowest haemoglobin, highest CRP, lowest neutrophil and WBC, lowest platelet count, lowest albumin, highest creatinine, highest ALT will be registered in the eCRF blood work.

§ Overall survival from HIPEC registry is also acquired prior to interim analysis
